# Comparison of elastography score and strain ratio values according to the presence of stress urinary incontinence in transperineal elastography

**DOI:** 10.3389/fmed.2026.1753479

**Published:** 2026-02-24

**Authors:** Can Ata, Ufuk Atlihan, Tevfik Berk Bildaci, Mehmet Emre Peker, Mehmet Ozer, Sercan Kantarci, Suna Yildirim Karaca, Alper Ileri

**Affiliations:** 1 Department of Obstetrics and Gynecology, Buca Seyfi Demirsoy Training and Research Hospital, Izmir Demokrasi University, Izmir, Türkiye; 2 Department of Obstetrics and Gynecology, Izmir Demokrasi University, Izmir, Türkiye; 3 Department of Obstetrics and Gynecology, Merkezefendi Hospital, Manisa, Türkiye; 4 Department of Obstetrics and Gynecology, University of Health Sciences Erzurum State Hospital, Erzurum, Türkiye; 5 Department of Obstetrics and Gynecology, Tepecik Training and Research Hospital, University of Health Sciences, Izmir, Türkiye; 6 Department of Obstetrics and Gynecology, Cigli Training and Research Hospital, Bakircay University, Izmir, Türkiye

**Keywords:** elastography score, levator ani muscle, pelvic floor, strain ratio, stress urinary incontinence

## Abstract

**Objective:**

To compare the elastography score (ES) and strain ratio (SR) values in transperineal elastography according to the presence of stress urinary incontinence (SUI).

**Materials and methods:**

This retrospective descriptive study included 72 women who underwent transperineal ultrasonographic evaluations of pelvic floor function between January 2020 and April 2022. The study group consisted of 32 women with SUI, and the control group included 40 continent women. Elastography assessments were performed by a single experienced gynecologist using strain elastography focused on the puborectalis portion of the levator ani muscle. The ES was graded on a four-point scale (1 = soft, 4 = hard), and SR values were calculated automatically as the ratio of reference soft tissue to levator ani muscle strain (SR = B/A).

**Results:**

The number of vaginal deliveries was significantly higher in the SUI group (2.4 ± 1.3 vs. 1.3 ± 0.9, *p* = 0.001). ES values were significantly lower in the SUI group compared with the controls at rest (ES: 1.9 ± 0.8 vs. 2.5 ± 0.8, *p* = 0.002). SR values were significantly lower in the SUI group compared with the controls at rest (SR: 1.72 ± 0.43 vs. 2.15 ± 0.47, *p* = 0.001). ES values were significantly lower in the SUI group compared with the controls during the Valsalva maneuver (ES: 2.6 ± 0.6 vs. 3.2 ± 0.5, *p* = 0.001). SR values were significantly lower in the SUI group compared with the controls during the Valsalva maneuver (SR: 2.94 ± 0.53 vs. 3.79 ± 0.61, *p* = 0.001).

**Conclusion:**

Transperineal elastography demonstrated a significant reduction in levator ani muscle elasticity in women with SUI. These findings suggest that SUI is an anatomic disorder that involves biomechanical dysfunction of pelvic floor tissues. Transperineal elastography may serve as a noninvasive, reproducible, and objective tool for evaluating pelvic floor muscle function and monitoring treatment outcomes in clinical practice.

## Introduction

Urinary incontinence (UI) is defined as the involuntary leakage of urine and represents a prevalent condition globally (1). UI is a common pelvic floor disorder, with stress urinary incontinence (SUI) affecting approximately 20–40% of women and significantly impairing quality of life (2, 3). SUI is characterized by involuntary leakage of urine during activities that increase intra-abdominal pressure, such as coughing, sneezing, or exercise. Pathophysiologically, it is associated with the loss of support from pelvic floor muscles, endopelvic fascia, and ligamentous structures (4, 5). Among the pelvic floor muscles, the levator ani muscle group plays the most critical role, contributing to the continence mechanism by maintaining the anatomic position of the urethra and vagina (6). Damage or reduced elasticity of the levator ani muscle is thought to contribute to urethral hypermobility and impaired continence mechanisms, particularly during increases in intra-abdominal pressure (7). Factors such as vaginal delivery, menopause, obesity, and chronic cough may increase the mechanical load on the levator ani muscle, leading to disruption of its structural integrity (8).

Traditional diagnostic methods, including clinical examination and urodynamic testing, reveal the functional aspects of SUI but do not provide information about the biomechanical properties of the pelvic floor muscles. Although transperineal ultrasonography allows assessment of pelvic floor anatomy, it does not provide objective information on tissue stiffness or elasticity, which are critical for continence function (9). Elastography, developed to address this limitation, is an ultrasound-based imaging technique that allows quantitative or semi-quantitative evaluation of tissue stiffness and elasticity. The principle of elastography is based on the fact that soft tissues undergo greater deformation under pressure, whereas stiffer tissues deform less (10). Elastography is an ultrasound-based technique that evaluates tissue stiffness based on deformation under applied pressure and has recently been applied to pelvic floor assessment (11, 12). As a result, physicians often lack objective, tissue-based parameters to explain symptoms or monitor treatment response in women with SUI.

Although previous studies have suggested altered elastic properties of the pelvic floor muscles in women with UI, data on standardized elastography parameters and their dynamic assessment remain limited (13). However, the number of studies on this subject remains limited, and further data are needed before the method can be fully integrated into clinical practice (14). The elastography score (ES) gives a semi-quantitative visual measure of tissue stiffness, and the strain ratio (SR) provides a relative quantitative comparison against reference soft tissues. However, there has been limited research on using both parameters together, under both resting and dynamic conditions. Moreover, the normal reference ranges and clinical correlations of elastography parameters such as ES and SR need to be validated in different populations. In this context, comparing the elasticity of the levator ani muscle between women with SUI and continent women may contribute to an objective evaluation of pelvic floor function.

However, despite growing interest in pelvic floor elastography, available data remain limited and heterogeneous. Most previous studies have focused on static measurements or single elastographic parameters, and the combined evaluation of ES and SR under both resting and dynamic conditions has not been sufficiently explored. Consequently, the clinical applicability of transperineal elastography in SUI remains to be clearly defined.

Therefore, the aim of this study was to compare ES and SR values of the levator ani muscle between women with SUI and continent controls using transperineal strain elastography at rest and during Valsalva maneuvers.

## Materials and methods

The present study had a retrospective observational design following the Principles of the Helsinki Declaration. Informed consent documents were received from all patients. The study received approval from University of Health Sciences, Tepecik Training and Research Hospital Ethics Committee (date: 25/05/2022, number: 2022/8/1). The study was conducted in the Obstetrics and Gynecology Clinic of University of Health Sciences, Tepecik Training and Research Hospital, a tertiary referral center, where pelvic floor ultrasonography and elastography are routinely performed by experienced gynecologists. The medical records of women who underwent pelvic floor function evaluations using transperineal ultrasonography in our Obstetrics and Gynecology Clinic between January 2020 and April 2022 were evaluated retrospectively. The inclusion criteria were as follows: age between 18 and 70 years, a diagnosis of SUI confirmed through clinical history, pelvic examination and/or urodynamic testing, and complete transperineal ultrasound and elastography data available in the archive. SUI was defined as the involuntary loss of urine through the urethra attributable to a sudden increase in intra-abdominal pressure (15). The Urinary Distress Inventory (UDI-6) and Incontinence Impact Questionnaire (IIQ-7) data of all patients were retrospectively reviewed (16). The control group consisted of women in the same age range with no history of UI or pelvic floor dysfunction, and with normal pelvic ultrasonography findings during routine examinations. The exclusion criteria included mixed or urge incontinence, pelvic organ prolapse stage ≥2, history of pelvic reconstructive or anti-incontinence surgery, neurologic disorders, active pelvic infection, pregnancy, or postpartum status within 6 months. To minimize selection bias, all consecutive women who met the inclusion criteria and underwent transperineal ultrasonography with elastography during the study period were included, and no selective sampling was performed. According to these criteria, 32 women with SUI were included in the study group, and 40 healthy women formed the control group. All elastography examinations were performed by the same experienced gynecologist.

**Figure 1:**
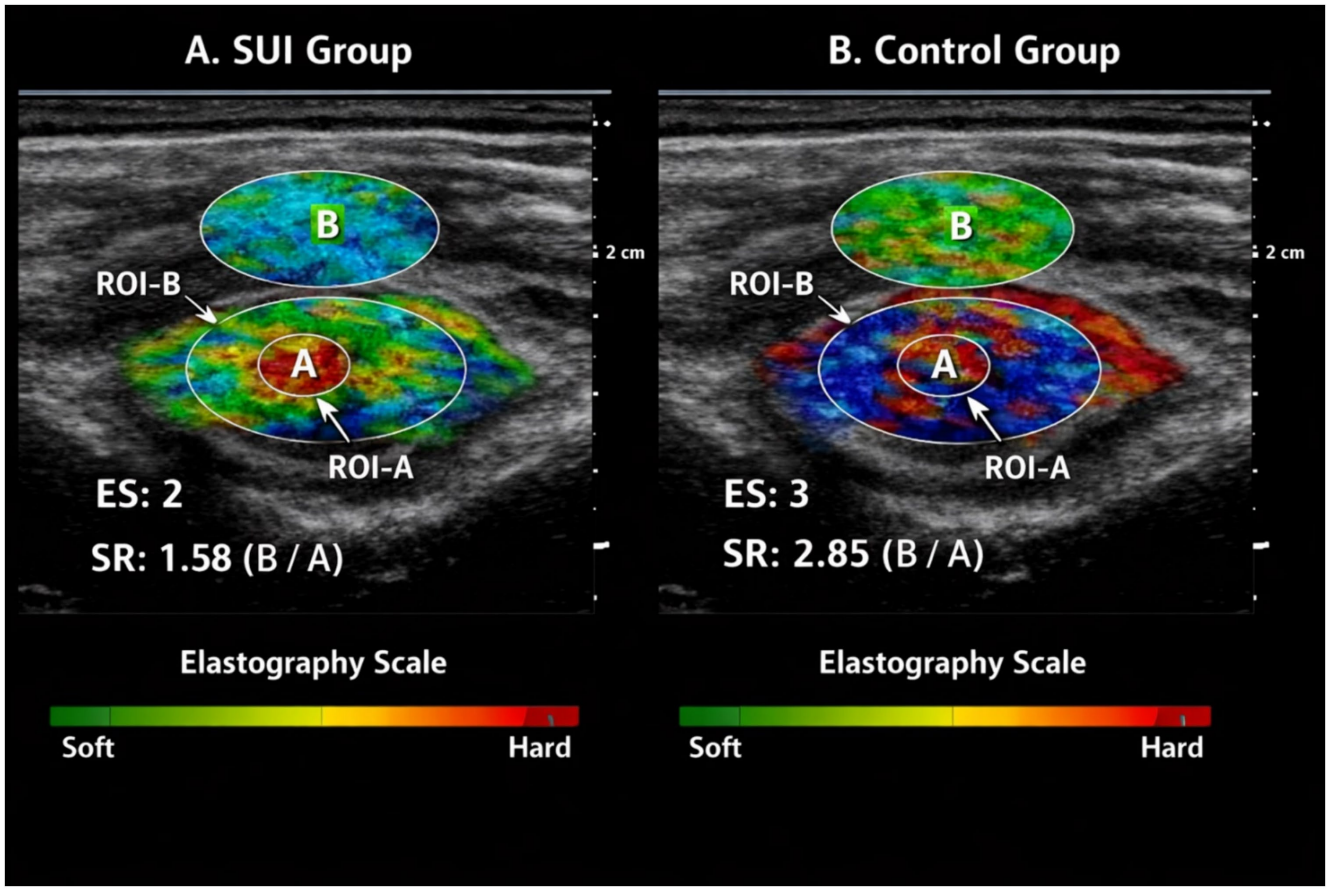
Representative transperineal strain elastography images. Representative transperineal strain elastography images of the puborectalis portion of the levator ani muscle **(A)**. A patient with SUI showing lower tissue stiffness (lower ES and SR values) **(B)**. A continent control subject demonstrating higher muscle stiffness. ROI-A indicates the levator ani muscle; ROI-B represents the reference soft tissue used for strain ratio calculation (SR = B/A).

Participants were placed in the lithotomy position, ensuring that they had urinated completely. Initially, conventional transperineal ultrasonography was used to visualize the bladder neck, urethra, and levator ani muscle. Subsequently, the strain elastography mode was activated, focusing on the puborectalis component of the levator ani. Gentle and rhythmic manual compression was applied to the probe, and the tissue deformation response was displayed as a color elastogram. Two main parameters were analyzed in the elastography assessment: ES and SR. ES reflects tissue stiffness in a semi-quantitative manner using a four-grade scale, where ES 1 indicates soft tissue (predominantly green-yellow colors), ES 2 moderately soft tissue, ES 3 moderately hard tissue, and ES 4 hard tissue (predominantly red-blue colors). Mean ES values were recorded both at rest and during the maximum Valsalva maneuvers. SR was automatically calculated using the ultrasound system using the formula *SR = B/A*, where region of interest-A (ROI-A) represented the levator ani muscle and region of interest-B (ROI-B) was a reference soft tissue, typically the perianal region, at the same depth. Each measurement was repeated three times, and the average of these measurements was used for statistical analysis (see Figure 1).

The primary outcome of the study was the difference in ES and SR values of the levator ani muscle between women with SUI and continent controls at rest and during Valsalva maneuvers. Secondary outcomes included the association between obstetric history (number of vaginal and cesarean deliveries) and elastography parameters, as well as differences in UDI-6 and IIQ-7 scores between the groups.

## Statistical analysis

Statistical analysis was performed using the SPSS version 26.0 software package (IBM Inc., Chicago, IL, United States). The normality of data distribution was assessed using the Kolmogorov–Smirnov test. Descriptive statistics for categorical variables are presented as percentages and frequencies, and descriptive statistics for numerical variables are expressed as mean, standard deviation, and minimum (min)–maximum (max) values. Student’s *t*-test was used for normally distributed variables, and the Mann–Whitney U test was used for non-normally distributed variables. The independent samples t-test was used to compare paired groups. Results were evaluated at 95% confidence intervals (CI). *p*-values of < 0.05 were considered statistically significant.

## Results

The number of vaginal deliveries was significantly higher in the SUI group compared with the control group (2.4 ± 1.3 vs. 1.3 ± 0.9; *p* = 0.001). Conversely, the number of cesarean deliveries was significantly lower in the SUI group (0.7 ± 0.6 vs. 1.2 ± 0.8; *p* = 0.003) (Table 1).

**Table 1:** Comparison of demographic and obstetric characteristics of the participants.

Variables	Control group (*n* = 40)	SUI group (*n* = 32)	*p*-value
Age (years)	51.6 ± 6.9	52.9 ± 8.2	0.48
Body mass index (kg/m^2^)	25.9 ± 2.2	26.7 ± 2.4	0.15
Gravida (median, IQR)	2 (2–3)	3 (2–4)	0.167
Parity (median, IQR)	2 (1–3)	3 (2–4)	0.118
Menopausal status (postmenopausal, *n* %)	15 (35.0%)	14 (40.6%)	0.768
Vaginal delivery (mean ± SD)	1.3 ± 0.9	2.4 ± 1.3	0.001
Cesarean delivery (mean ± SD)	1.2 ± 0.8	0.7 ± 0.6	0.003

In the SUI group, the ES values measured both at rest and during maximum Valsalva maneuvers were significantly lower compared with the control group (at rest: 1.9 ± 0.8 vs. 2.5 ± 0.8, *p* = 0.002; maximum Valsalva: 2.6 ± 0.6 vs. 3.2 ± 0.5, *p* = 0.001). Similarly, the SR values were also significantly lower in the SUI group both at rest and during Valsalva maneuvers (at rest: 1.72 ± 0.43 vs. 2.15 ± 0.47, *p* = 0.001; maximum Valsalva: 2.94 ± 0.53 vs. 3.79 ± 0.61, *p* = 0.001) (Table 2).

**Table 2:** Comparison of elastography parameters between the control and stress urinary incontinence groups.

Variables		Control group (*n* = 40)	SUI group (*n* = 32)	*p*-value
		(Mean ± SD)		
Elastography score	At rest	2.5 ± 0.8	1.9 ± 0.8	0.002
	Maximum Valsalva	3.2 ± 0.5	2.6 ± 0.6	0.001
Strain ratio	At rest	2.15 ± 0.47	1.72 ± 0.43	0.001
	Maximum Valsalva	3.79 ± 0.61	2.94 ± 0.53	0.001

The UDI-6 score was significantly lower (24.72 ± 11.98) in the control group compared with the SUI group (50.88 ± 17.76) (*p* < 0.001). The IIQ-7 score was significantly lower (24.56 ± 11.68) in the control group compared with the SUI group (48.22 ± 18.96) (*p* < 0.001) (Table 3).

**Table 3:** Comparison of incontinence scores between groups.

Variables	Control group (*n* = 40)	SUI group (*n* = 32)	*p*-value
	Mean ± SD		
UDI-6	24.72 ± 11.98	50.88 ± 17.76	<0.001
IIQ-7	24.56 ± 11.68	48.22 ± 18.96	<0.001

UDI-6: Urinary distress inventory, IIQ-7: Incontinence impact questionnaire.

## Discussion

The main finding of this study is that women with SUI had significantly lower ES and SR values of the levator ani muscle compared with continent women. This difference was observed both at rest and during Valsalva maneuvers. These findings indicate reduced levator ani muscle stiffness in women with SUI and suggest impaired biomechanical support of the pelvic floor. Also, that the levator ani muscle in patients with SUI has a softer structure and, consequently, a diminished capacity to provide urethral support. Such alterations in the mechanical properties of the pelvic floor muscles have been reported to play an important role in the pathophysiology of SUI, as emphasized by Bø and Sherburn (17) and Ashton-Miller et al. (18).

Pelvic floor function is closely related to anatomic integrity, muscle tone, and tissue elasticity. The tonic contraction of the levator ani muscle is particularly critical for maintaining urethral closure pressure (19). Loss of elasticity in muscle tissue may occur as a result of childbirth-related trauma or chronic mechanical loading, leading to weakening of the urethral support mechanism (20). In our study, the higher number of vaginal deliveries in the SUI group was consistent with the findings reported by Rortveit et al. and Sultan et al., who demonstrated the adverse effects of vaginal delivery on the structural integrity of pelvic floor muscles in postpartum women (21, 22).

Although transperineal ultrasonography is widely used to evaluate pelvic floor anatomy, its ability to objectively assess muscle stiffness and elasticity is limited. Elastography is an advanced imaging modality that addresses this limitation by enabling evaluation of the mechanical response of soft tissues (23). Previous studies in this field have shown that pelvic floor muscle stiffness measured by elastography in the postpartum period can predict the presence of symptomatic incontinence, as reported by Khandheria et al. (24). Similarly, Dietz and Shek demonstrated that decreased levator ani muscle elasticity was evident in both stress and mixed urinary incontinence (25). In addition, the study by Yu et al. (26) revealed significantly lower elastography parameters in patients with SUI. Our findings are consistent with these studies and demonstrate that deterioration in the elastic properties of the levator ani muscle can be assessed under both resting and dynamic conditions.

One of the distinctive aspects of this study is the evaluation of levator ani muscle elasticity both at rest and during maximal Valsalva maneuvers. This approach provides information on tonic muscle properties and dynamic and reflex response capacity. Furthermore, our study includes a wider age range compared with many previous reports in the literature. Nevertheless, standardization of elastography measurements has not yet been fully established, and factors such as device type, probe pressure, and ROI selection may influence the results (27). The fact that all measurements were performed by the same experienced gynecologist helped to reduce this variability.

Recent studies have demonstrated a close association between pelvic floor muscle elasticity, mode of delivery, and the presence of SUI. Okada et al. (28) reported that levator ani muscle elasticity varied according to delivery mode and that the muscle became significantly softer after vaginal delivery. Similarly, Li et al. (29) showed that the soft tissue characteristics of the perineal body were significantly reduced in women with SUI. In the study by Okçu et al. (30), elastography was highlighted as having high accuracy in detecting differences in pelvic floor muscle stiffness in women with SUI. Moreover, Csákány et al. (31) suggested that sonoelastography could be used to map weak areas of the pelvic floor and might be valuable for the early detection of SUI.

The strengths of our study include a relatively large sample size and the simultaneous evaluation of ES and SR values; however, the retrospective design and single-center setting constitute limitations. In addition, the reproducibility and interobserver variability of elastography measurements were not assessed, and potential confounding factors such as hormonal status, level of physical activity, body mass index, and menopausal status could not be fully controlled. Therefore, prospective multicenter studies using standardized elastography protocols are needed to confirm and expand upon these findings.

## Conclusion

Our study demonstrates a significant reduction in the elasticity of the levator ani muscle among women with SUI. This finding suggests that SUI is both an anatomic disorder and a functional problem related to tissue biomechanics. Transperineal elastography may serve as a noninvasive, reproducible, and objective indicator of pelvic floor muscle function that can be integrated into clinical practice. Future large-scale, prospective, and multicenter studies are warranted to further clarify the diagnostic and prognostic value of this method.

## Data Availability

The original contributions presented in the study are included in the article/supplementary material, further inquiries can be directed to the corresponding author.
